# Awareness, perception and perpetration of cyberbullying by high school students and undergraduates in Thailand

**DOI:** 10.1371/journal.pone.0267702

**Published:** 2022-04-29

**Authors:** Salinee Thumronglaohapun, Benchalak Maneeton, Narong Maneeton, Sasikarn Limpiti, Natthaporn Manojai, Jeerayut Chaijaruwanich, Unyamanee Kummaraka, Ruethaichanok Kardkasem, Tanarat Muangmool, Suttipong Kawilapat, Kanokkarn Juntaping, Patrinee Traisathit, Pimwarat Srikummoon

**Affiliations:** 1 Department of Statistics, Faculty of Science, Chiang Mai University, Chiang Mai, Thailand; 2 Department of Statistics, Faculty of Science, Data Science Research Center, Chiang Mai University, Chiang Mai, Thailand; 3 Department of Psychiatry, Faculty of Medicine, Chiang Mai University, Chiang Mai, Thailand; 4 Faculty of Mass Communication, Chiang Mai University, Chiang Mai, Thailand; 5 Mplus Foundation, Chiang Mai, Thailand; 6 Department of Computer Science, Faculty of Science, Data Science Research Center, Chiang Mai University, Chiang Mai, Thailand; 7 Department of Obstetrics and Gynecology, Faculty of Medicine, Chiang Mai University, Chiang Mai, Thailand; 8 Research Center in Bioresources for Agriculture, Industry and Medicine, Chiang Mai University, Chiang Mai, Thailand; SUNY Downstate Health Sciences University, UNITED STATES

## Abstract

The modern online society requires everyone, especially children and young people, to learn how to use the Internet. Cyberbullying is one misuse that can be detrimental to the cyberbullied individuals’ mental health and lifestyle, and it often ends up with the victim becoming depressed, fearful of society, and in the worst cases, suicidal ideation. The aim of this study is to investigate the awareness, perception, and perpetration of cyberbullying by high school students and undergraduates to find ways to prevent cyberbullying in the future. For this cross-sectional study, data were collected in 2020 from 14 schools throughout Thailand and 4 universities in Chiang Mai, Thailand, using two-stage sampling. Chi-squared tests were used to compare differences between the groups. Of the 2,683 high school students, girls perceived cyberbullying more than boys (81.6% vs. 75.4%; *p* <0.001), with those from the later academic years being more aware of cyberbullying (*p* = 0.033) and more likely to conduct cyberbullying behavior (*p* = 0.027). Of the 721 undergraduates, women were more aware of cyberbullying than men (92.1% vs. 82.7%; *p* <0.001). The most common cause of cyberbullying was aiming to tease the target (67.6% of high school students vs. 82.5% of undergraduates). The most commonly cyberbullying victimization was sending mocking or rebuking messages (29.6% of high school students and 39.6% of undergraduates). The most popular solutions for cyberbullying were to avoid leaving a trace on social media and be with friends who accept who you are. Our findings show that most of the cyberbullying perpetrators did not consider that their actions would have serious consequences and only carried out cyberbullying because of wanting to tease their victims. This is useful information for the cyberbullying solution center, teachers, and parents to recognize how to make the students realize the effects of cyberbullying on the victims.

## Introduction

Since the world has already entered the digital information age, online social networking platforms have grown to exact influence on society, especially in Thailand with 72% of Internet users or approximately 50.1 million of the total Thai population accessing them [1]. In 2020, it is estimated that 98.4% of adolescents (aged 15–24 years) use the Internet [2]. It has been reported that the prevalence of cyberbullying among US and Australian adolescents is 4.5% and 72%, respectively [3, 4], while in Thailand, the prevalence is 39.0–70.7% [5, 6].

According to the findings from previous studies, factors related to cyberbullying victimization and perpetration include demographic characteristics such as gender [7–15] and age [16–18], Internet usage or pattern [8, 12, 18, 19], and cyberbullying pattern and role [9–14, 16–24]. Furthermore, the results from a recent study in South Korea show that white female adolescents are more likely to be cyberbullied [25].

Psychological factors such as low self-esteem negatively affect the victim whereas perpetrators bully due to frustration, moral abandonment, sadism, lack of empathy, and ignoring other people [10, 12, 24, 26–34]. Not only personal factors but also parental characteristics such as parental education level and parenting style are related to cyberbullying from both victim’s and perpetrator’s perspectives [11, 18, 35, 36]. Cyberbullying victims can experience psychological and emotional trauma such as depression, anxiety, and suicidal ideation [4]. Nevertheless, further social and academic studies are needed to provide both short-term and long-term solutions for the effective reduction or prevention of the problems associated with cyberbullying [37–41].

It is important to investigate the students’ awareness and perception of cyberbullying to appropriately use the information and communication technologies [42]. The students need to be informed about the harm cyberbullying can do to the victim and how perpetration of it can be traced [43]. Thus, increasing cyberbullying awareness would reduce the likelihood of individuals perpetrating it [44]. Elçi and Seçkin found that female students have significantly less awareness than males [45]. Huang and Chou reported 63.4% of Taiwanese students have perceived cyberbullying, with 34.9% being victims and 20.4% being perpetrators [46]. Ozden and Icellioglu found that male undergraduates were significantly more likely to be cyberbullies than females. In addition, more male undergraduates perceived that cyberbullying behavior was harmless than females [47].

Although researchers in several previous studies have focused on cyberbullying among young people, none have clearly compared its presence and impact on high school and undergraduate students as separate groups [12, 16, 18]. Living with parents is one of the predictive factors affecting cyberbullying from both the victims’ and perpetrators’ perspectives [11, 18, 35, 36]. However, this may not be relevant for undergraduate students living alone who must manage the problem without parental guidance. With or without parental support, high school student cyberbullying victims are often unable to manage their problems, which can end up in self-harm or suicide attempts [37]. The transition from high school to university is an important stage in life offering the potential for personal growth and behavioral change [48]. The results from a survey about cyberbullying among students in universities and high schools across Turkey found that behavioral and emotional reactions toward cyberbullying varied according to school level [49]. Although the learning behaviors of high school students and graduate students are separated by age, education level, and life experience, comparing the similarities and differences in cyberbullying behavior between these two groups could help to uncover trends in cyberbullying behavior during the educational process of young people that may lead to better remedies for avoiding or mitigating this behavior.

Social networks are changing so rapidly that accompanying research is necessary to keep up to date. The awareness, perception, and perpetration of cyberbullying using the Internet are also likely to change as social networks evolve. The aim of the present study is to investigate the awareness, perception, and perpetration of cyberbullying and its associated factors among high school students and undergraduates to find ways to prevent it in the future.

## Methods

### Participants and setting

In this cross-sectional study, we collected data separately from high schools nationwide and universities in Chiang Mai, Thailand, between May and August 2020. The sample size of 2,750 high school students was preliminarily determined using a simple random sampling method on representative nationwide medium-to-high education level schools with ≥300 high school students, including 6 in northern Thailand, 3 in central Thailand, 2 in eastern Thailand, 2 in northeastern Thailand, and 1 in southern Thailand. The number of classrooms and classroom level were not considered in this randomization scheme to simplify the process and reduce the problems associated with remote data collection. The responsibility for data collection was assigned to a staff member at each school to complete the sampling according to the target number.

Two-stage sampling of undergraduates was performed at universities in Chiang Mai province, Thailand. The first step was to divide the 8 universities by randomly selecting 4 of them. The second step was to divide the sample according to the field of study by randomly selecting study plans from each university (Science and Technology, Arts and Social Sciences, and Health Sciences faculties) except for the Chiang Mai Campus, Thailand National Sports University, which was not classified by subject area.

### Data collection procedures

Accessing or requesting information was carried out by contacting the school or university staff. The questionnaires, consent forms, and research project essentials related to the study were sent by mail. The envelopes were sealed and only the responsible person distributed the questionnaires. The participants received a consent form and questionnaire from the responsible staff member at each school/university and filled in the questionnaire at their convenience at their school/university and/or home. Parental consent was required for participants who were under 18 years of age before completing the questionnaire. The questionnaires were anonymized and gathered by the responsible staff members and sent back to the principal investigators.

### Questionnaire

The questionnaire was developed and adapted to the Thai context based on the Coping with Cyberbullying Questionnaire (CWCBQ), the European Cyberbullying Intervention Project Questionnaire (ECIPQ), and a survey on bullying behavior indicators in adolescents in the Songkhla province, Thailand [50–52]. It was also evaluated for reliability and validity by using Cronbach’s alpha, exploratory factor analysis (EFA), and confirmatory factor analysis (CFA). This questionnaire consisted of three parts, including (1) demographics of the participants, (2) cyberbullying victimization, and (3) dealing with cyberbullying. In the first part, the items related to the demographic of participants consisted of 15 items including gender, class, people in the participant’s residence, the province where you come from, frequency of spending time with the family, family income, usage of social media applications, frequency of application usage, devices used to access online media, cyberbullying awareness, and cyberbullying awareness channels. This section of the questionnaire included single-choice and multiple-choice items.

There were 12 items related to cyberbullying actions, including “repeatedly sending disruptive messages”, “posting threatening messages”, “posting pictures to cause shame”, etc. according to the following frequency ranges: 0, 1–5, 6–10, 11–15, and >15 times /year.

The question related to the awareness of cyberbullying, “Do you know what cyberbullying is?”, required a response of “know” or “do not know”. The question related to the perception of cyberbullying was “Have you ever seen or experienced cyberbullying?” and required the response of “never experienced” or “experienced” (the meaning of “experienced” in Thai can be interpreted as “yes” in English). The perpetration of cyberbullying was asked as “Have you ever cyberbullied someone?”, with answers of “ever” or “never”. More information related to cyberbullying including the channels of cyberbullying awareness, the list of cyberbullying actions, and the reaction to cyberbullying were in the following sections. Participants who knew what cyberbullying is were asked about how they became aware (i.e., the channel of cyberbullying awareness). They could select multiple answers, including “social media”, “own experience”, “teacher/school/training”, and “other, please specify". Participants who had seen or experienced cyberbullying were asked about their actions toward it. They could select multiple answers, including “ignore/avoid”, “dissuade the perpetrators”, “join the perpetration”, “inform teachers/parents”, “collect evidence for the victim”, and "other, please specify”. Participants who had perpetrated cyberbullying were asked about their reasons for doing so. They could select multiple answers, such as “aiming to tease someone”, “having previously been a victim”, “revenge on previous perpetrators”, “coerced by other people”, “dislike of the victim”, “defamation by the victim”, etc.

The third part related to dealing with cyberbullying comprised 19 items, such as “ignore the person who is cyberbullying”, “try not to think about being teased/ cyberbullied”, “block the cyberbully”, “avoid posting private information”, “avoiding leaving traces on online media such as passwords”, “inform family members/adults/trusted people about the cyberbullying”, “discuss the cyberbullying with a friend or trusted person”, “spending time with friends who accept you for who you are”, “seek advice on social media”, “seek advice from someone who has experienced cyberbullying”, “seek help from a parent/teacher”, “seek help from other people such as police/psychiatrists”, “inform the service provider to stop publishing the content”,”review privacy settings for online comments”, “collect evidence of cyberbullying for retaliation”, “tell the cyberbullies to stop”, “refuse to forward messages or images that hurt the victim”, “help collect evidence and notify people who have the potential to help” and “discourage the cyberbullying perpetrator(s)” with responses in the form of three frequencies of action: never, sometimes, or always.

The properties of cyberbullying victimization and reaction to cyberbullying scales were evaluated using Cronbach’s alpha, EFA, and CFA, respectively. Cronbach’s alpha >0.70 indicates that the item has acceptable reliability [53]. The number of factors considered from the factor with eigenvalue >1 in EFA [54]. The indices according to CFA including a comparative fit index (CFI) of >0.90, a Tucker Lewis index (TLI) of >0.90, and a root-mean-squared error of approximation (RMSEA) of <0.10 indicate that the fitting of the scale was adequate [55].

### Outcomes

The outcomes of the study were measuring the participants’ awareness, perception, and perpetration of cyberbullying via binary responses to items concerning cyberbullying, including (1) “Do you know what cyberbullying is?” to ascertain their awareness, (2) “Have you ever been cyberbullied yourself?” to ascertain their experiences of being the victim, and (3) “Have you ever cyberbullied others?” to ascertain their experiences of being the perpetrator.

### Statistical analysis

Descriptive statistics are presented as frequencies and percentages for categorical data and the median and interquartile ranges for continuous data. We compared three cases between the two groups: with/without awareness of cyberbullying, with/without perception of cyberbullying, and have/have not been subjected to cyberbullying behavior using Chi-squared tests for the categorical variables and Mann-Whitney U tests for the continuous variables. Fisher’s exact test was additionally used for variables that violated the assumption of expected frequency in the Chi-squared test. All analyses were performed using Stata 17. Statistical significance for all analyses was set as *p* ≤ 0.05.

### Ethical approval

The study protocol abides by the Declaration of Helsinki and was reviewed and approved by the Chiang Mai University Research Ethics Committee (CMUREC No. 26/161). Written informed consent was obtained from all participants or their parents after receiving an explanation of the study.

## Results

### Properties of the cyberbullying victimization and reaction to cyberbullying scales

The internal consistency of the cyberbullying victimization scale was acceptable for all items (a Cronbach’s alpha coefficient range of 0.8509–0.8616). According to the EFA results, all of the 12 items were retained as one factor and were fitted for measuring cyberbullying victimization (CFI = 0.876, TLI = 0.849, and RMSEA = 0.097) (S1–S3 Tables).

All of the items in the reaction to cyberbullying scale had acceptable internal consistency (a Cronbach’s alpha coefficient range of 0.8532–0.8638). According to the EFA results, the items were separated into two subscales: (1) seeking support from other people (items 6, 7, 9, 10, 11, 12, 13, 15, 16, 18, and 19) and (2) disregarding and preventing cyberbullying (items 1, 2, 3, 4, 5, 8, 14, and 17). The index values of the two subscales were slightly lower than those in the CFI and TLI criteria and slightly higher than that of the RMSEA criterion (CFI = 0.792, TLI = 0.740, and RMSEA = 0.123 for the seeking support from other people subscale and CFI = 0.866, TLI = 0.812, and RMSEA = 0.108 for the disregarding and prevention of cyberbullying subscale) (S4–S6 Tables).

### Demographics of the high school students

Of the 2,683 high school students who completed the questionnaire, the dropout rate was 2.43%, most of the respondents were female (69.5%), and the median age was 16 years old (interquartile range (IQR): 16–17). We found that the most used social media platforms were Facebook, Instagram, and YouTube, accounting for 32%, 31.2%, and 25.6%, respectively (Table 1).

**Table 1:** Demographic data and awareness, perception, and perpetration of cyberbullying by high school students in Thailand.

Variable (Number (%^a^) or Median [IQR])	Total	Awareness			Perception			Perpetration		
		No	Yes	*P* ^b^	No	Yes	*P* ^b^	No	Yes	*P* ^b^
**Gender**				<0.001			<0.001			0.902
Male	817 (30.5)	174 (21.5)	634 (78.5)		196 (24.6)	602 (75.4)		619 (78.6)	169 (21.4)	
Female	1,864 (69.5)	291 (15.9)	1,545 (84.1)		337 (18.4)	1,495 (81.6)		1,421 (78.8)	383 (21.2)	
**Educational year**				0.033			0.135			0.068
Grade 10	750 (28)	153 (20.6)	588 (79.4)		167 (22.8)	567 (77.2)		584 (81.2)	135 (18.8)	
Grade 11	1,097 (40.9)	181 (16.8)	898 (83.2)		210 (19.4)	872 (80.6)		837 (78.7)	226 (21.3)	
Grade 12	832 (31.1)	131 (15.9)	691 (84.1)		155 (19.1)	657 (80.9)		617 (76.4)	191 (23.6)	
**Age (years)**	16 [16–17]	16 [16–17]	16 [16–17]	0.736	16 [16–17]	16 [16–17]	0.509	16 [16–17]	17 [16–17]	0.027
**Body weight (kg)**	53 [47–61]	52.5 [47–60]	53 [47–61]	0.262	53 [46–60]	53 [[47–61]	0.522	53 [46–60.7]	53 [47–62]	0.304
**Height (cm)**	163 [158–169]	164 [158–170]	163 [158–169]	0.068	164 [158–169]	163 [158–169]	0.059	163 [158–169]	163 [158–163]	0.400
**Region**				<0.001			<0.001			<0.001
Northern	869 (32.4)	79 (9.2)	784 (90.8)		145 (17)	709 (83)		614 (72.7)	231 (27.3)	
Central	716 (36.7)	133 (19)	569 (81)		116 (16.6)	585 (83.4)		530 (77.2)	157 (22.8)	
Eastern	390 (14.5)	60 (15.4)	330 (84.6)		90 (23.2)	298 (76.8)		308 (81)	72 (19)	
Northeastern	444 (16.6)	154 (35.6)	278 (64.4)		112 (25.9)	320 (74.1)		367 (86)	60 (14)	
Southern	264 (9.8)	39 (15.1)	220 (84.9)		70 (27.2)	187 (72.8)		223 (87.4)	32 (12.6)	
**History of Moving School**				0.434			0.043			0.471
No	2,307 (87.2)	395 (17.4)	1,880 (82.6)		471 (20.8)	1,794 (79.2)		1,767 (78.9)	473 (21.1)	
Yes	338 (12.8)	64 (19.1)	271 (80.9)		53 (16.0)	278 (84)		246 (77.1)	73 (22.9)	
**Relationship status**				0.046			0.041			0.511
Single	2,422 (90.9)	409 (17.1)	1,983 (82.9)		494 (20.8)	1,882 (79.2)		1,847 (78.9)	495 (21.1)	
With a partner	242 (9.1)	53 (22.3)	185 (77.7)		36 (15.2)	201 (84.8)		181 (77)	54 (23)	
**Religion**				0.135			0.027			0.013
Buddhism	2,420 (91.4)	430 (18)	1,960 (82)		481 (20.3)	1,892 (79.7)		1,839 (78.6)	500 (21.4)	
Christianity	56 (2.1)	5 (8.9)	51 (91.1)		4 (7.1)	52 (92.9)		37 (66.1)	19 (33.9)	
Islam	163 (6.2)	24 (15.1)	135 (84.9)		37 (23.3)	122 (76.7)		134 (85.9)	22 (14.1)	
Others	9 (0.3)	0 (0)	8 (100)		0 (0)	9 (100)		8 (88.9)	1 (11.1)	
**Living status**				0.027			0.006			0.006
Both parents	1,746 (65.4)	317 (18.4)	1,405 (81.6)		378 (22.1)	1,335 (77.9)		1,368 (80.5)	331 (19.5)	
Father or mother	568 (21.3)	77 (13.8)	483 (86.2)		82 (14.7)	477 (85.3)		405 (75.1)	134 (24.9)	
Other relatives	321 (12)	64 (20.2)	252 (79.8)		66 (21.1)	246 (78.9)		238 (76.5)	73 (23.5)	
Alone	13 (0.5)	0 (0)	13 (100)		2 (15.4)	11 (84.6)		6 (50)	6 (50)	
Not with relatives	20 (0.8)	3 (15)	17 (85)		4 (20)	16 (80)		15 (75)	5 (25)	
**Family time activity (hours/week)**				0.032			0.053			<0.001
< 2	270 (10.1)	47 (17.8)	217 (82.2)		50 (19.1)	212 (80.9)		186 (72.1)	72 (27.9)	
2–5	833 (31.1)	138 (16.7)	689 (83.3)		149 (18.3)	666 (81.7)		608 (75.5)	197 (24.5)	
6–10	660 (24.7)	139 (21.3)	513 (78.7)		156 (23.9)	496 (76.1)		531 (83.2)	107 (16.8)	
> 10	912 (34.1)	141 (15.8)	754 (84.2)		178 (19.9)	717 (80.1)		711 (80.3)	174 (19.7)	
**Average monthly family income (baht)**				<0.001			0.002			0.281
< 10,000	475 (17.9)	124 (26.8)	339 (73.2)		120 (25.9)	344 (74.1)		367 (81.7)	82 (18.3)	
10,001–20,000	1,060 (39.9)	190 (18.2)	855 (81.8)		215 (20.7)	824 (79.3)		810 (79.1)	214 (20.9)	
20,001–30,000	564 (21.3)	97 (14.1)	480 (85.9)		92 (16.6)	462 (83.4)		426 (77.7)	122 (22.3)	
> 30,000	554 (20.9)	71 (12.9)	478 (87.1)		102 (18.7)	443 (81.3)		418 (77)	125 (23)	
**Social media platform**				<0.001			<0.001			0.027
Facebook	714 (32.0)	199 (28.2)	506 (71.8)		168 (24.2)	527 (75.8)		557 (80)	139 (20)	
Line	39 (1.7)	5 (13.2)	33 (86.8)		7 (18.4)	31 (81.6)		31 (86.1)	5 (13.9)	
Instagram	695 (31.2)	86 (12.6)	598 (87.4)		109 (16)	573 (84)		523 (78.2)	146 (21.8)	
YouTube	571 (25.6)	100 (17.7)	464 (82.3)		194 (25.7)	417 (74.3)		447 (81.7)	100 (18.3)	
Twitter	183 (8.2)	6 (3.3)	176 (96.7)		16 (8.9)	164 (91.1)		133 (74.7)	45 (25.3)	
Others	29 (1.3)	4 (14.8)	23 (85.2)		6 (20.7)	23 (79.3)		18 (64.3)	10 (35.7)	
**Social media usage (hours/day)**				<0.001			<0.001			<0.001
< 2	95 (3.5)	26 (27.7)	68 (72.3)		34 (36.6)	59 (63.4)		81 (88)	11 (12)	
2–4	764 (28.5)	160 (21.4)	589 (78.6)		166 (22.3)	577 (77.7)		605 (81.8)	135 (8.2)	
5–6	934 (34.9)	166 (17.9)	760 (82.1)		185 (20.1)	736 (79.9)		721 (80.2)	178 (19.8)	
> 6	887 (33.1)	111 (12.7)	763 (87.3)		147 (16.8)	726 (83.2)		632 (73.5)	228 (26.5)	
**Devices used to access social media**										
**Private laptop/computer**	1,257 (53)			<0.001			0.001			0.262
No		316 (22.7)	1,076 (77.3)		315 (22.8)	1,069 (77.2)		1,079 (79.5)	278 (20.5)	
Yes		148 (11.8)	1,105 (88.2)		217 (17.5)	1,022 (82.5)		955 (77.7)	274 (22.3)	
**Public computer**	61 (23)			0.478			0.838			0.115
No		457 (17.6)	2,132 (82.4)		519 (20.2)	2,044 (79.8)		1,992 (78.9)	534 (21.1)	
Yes		8 (14)	49 (86)		13 (21.3)	48 (78.7)		43 (70.5)	18 (29.5)	
**Smartphone**	2,586 (96.8)			0.098			0.002			0.940
No		20 (24.4)	62 (75.6)		28 (33.7)	55 (66.3)		65 (78.3)	18 (21.7)	
Yes		444 (17.3)	2,119 (82.7)		503 (19.8)	2,091 (80.2)		1,968 (78.7)	534 (21.3)	
**Tablet**	237 (8.9)			<0.001			0.003			0.030
No		442 (18.4)	1,967 (81.7)		500 (20.9)	1,889 (79.1)		1,865 (79.2)	490 (20.8)	
Yes		23 (9.7)	214 (90.3)		30 (12.9)	203 (87.1)		168 (73)	62 (27)	

^a^ percentage of the total ware represented by column and percentage of the Awareness, Perception, and Perpetration of Cyberbullying were presented by row.

^b^ Comparisons were made by using Chi-squared tests for categorical variables and Mann-Whitney U tests for continuous variables. IQR, interquartile range.

### High school students’ awareness of cyberbullying

Girls had a higher awareness of cyberbullying than boys (84.1% vs. 78.5%; *p* <0.001). The proportion of cyberbullying awareness also increased with academic year (Grade 10 = 79.4% vs. Grade 11 = 83.2% vs. Grade 12 = 84.1%; *p* = 0.033). Students living alone were the most aware of cyberbullying, followed by living with a parent(s) (100% vs. 86.2%; *p* = 0.027). Spending more time on social media also increased awareness of cyberbullying (<2 hours = 72.3% vs. 2–4 hours = 78.6% vs. 5–6 hours = 82.1% vs. >6 hours = 87.3%; *p* <0.001) (Table 1).

### High school students’ perception of cyberbullying

We found that girls perceived cyberbullying more often than boys (81.6% vs. 75.4%; *p* <0.001). Students living with a parent(s) had a higher perception of cyberbullying than those living alone (85.3% vs. 84.6%; *p* = 0.006). Those who spent more time on social media had a greater perception of cyberbullying (<2 hours = 63.4% vs. 2–4 hours = 77.7% vs. 5–6 hours = 79.9% vs. >6 hours = 83.2%; *p* <0.001) (Table 1).

### High school students’ perpetration of cyberbullying

Cyberbullying perpetration increased with age (*p* = 0.027) and occurred more often when living alone or with someone other than a relative than when living with a parent(s) (50% vs. 25% vs. 19.5%; *p* = 0.006). Meanwhile, taking part in fewer family activities accounted for a higher incidence of cyberbullying behavior (<2 hours = 27.9% vs. 2–5 hours = 24.5% vs. 6–10 hours = 16.8% vs. >10 hours = 19.7%; *p* <0.001). The most commonly used devices for cyberbullying were tablets compared to others (27% vs. 20.8%; *p* = 0.030) (Table 1).

### Demographics of the undergraduates

Of the 721 respondents, the median age was 19 years old (IQR: 19–21) and 53% of them were female. 41.9%, 29.6%, 12.7%, 15%, and 0.8% of the respondents were from years 1, 2, 3, 4, and 5 or higher, respectively. Those who were from provinces other than Chiang Mai comprised 52.4%. The top three social media platforms accessed were Instagram, Facebook, and YouTube, accounting for 33.6%, 32.7%, and 14.9%, respectively (Table 2).

**Table 2:** Demographic data and awareness, perception, and perpetration of cyberbullying by undergraduates in Chiang Mai, Thailand.

Variable (Number (%^a^) or Median [IQR])	Total	Awareness			Perception			Perpetration		
		No	Yes	*p* ^*b*^	No	Yes	*p* ^*b*^	No	Yes	*p* ^*b*^
**Gender**				<0.001			0.204			0.003
Male	339 (47)	58 (17.3)	278 (82.7)		66 (20)	264 (80)		200 (60.1)	133 (39.9)	
Female	382 (53)	30 (7.9)	348 (92.1)		61 (16.3)	313 (83.7)		264 (70.6)	110 (29.4)	
**Educational year**				0.288			0.734			0.020
1st year	301 (41.9)	39 (13.1)	258 (86.9)		58 (19.9)	234 (80.1)		196 (66.2)	100 (33.8)	
2nd year	213 (29.6)	26 (12.3)	185 (87.7)		34 (16.3)	175 (83.7)		147 (70.3)	62 (29.7)	
3rd year	91 (12.7)	15 (16.7)	75 (83.3)		18 (20.2)	71 (79.8)		57 (65.5)	30 (34.5)	
4th year	108 (15)	8 (7.4)	100 (92.6)		16 (15.1)	90 (84.9)		62 (57.4)	46 (42.6)	
≥5th year	6 (0.8)	0 (0)	6 (100)		1 (16.7)	5 (83.3)		1 (16.7)	5 (83.3)	
**Age (years)**	19 [19–21]	19 [19–21]	19 [19–21]	0.975	19 [19–21]	19 [19–21]	0.576	19 [19–21]	19 [19–21]	0.086
**Bodyweight**	56 [49–65]	59 [50–66.7]	55 [49–65]	0.140	58 [50–65]	55 [49–65]	0.098	55 [49–65]	58 [50.3–65]	0.007
**Height**	165 [159–171]	170 [160–173]	165 [159–171]	0.030	169 [162–173]	165 [158–171]	0.001	165 [159–171]	165 [160–171]	0.267
**Usual residence**				<0.001			0.005			0.872
Chiang Mai	340 (47.6)	56 (16.7)	279 (83.3)		74 (22.4)	257 (77.6)		220 (66.1)	113 (33.9)	
Others	375 (52.4)	31(8.3)	342 (91.7)		52 (14.1)	316 (85.9)		241 (65.5)	127 (34.5)	
**Relationship status**				0.138			0.057			0.804
Single	586 (81.6)	76 (13.1)	504 (86.9)		111 (19.4)	460 (80.6)		374 (65.3)	199 (34.7)	
With a partner	132 (18.4)	11 (8.4)	120 (91.6)		16 (12.3)	114 (87.7)		87 (66.4)	44 (33.6)	
**Religion**				0.018^c^			0.246^c^			0.258^c^
Buddhism	634 (90)	70 (11.2)	558 (88.8)		111 (17.9)	510 (82.1)		418 (67.1)	205 (32.9)	
Christianity	49 (7)	13 (27.1)	35 (72.9)		11 (23.4)	36 (76.6)		27 (55.1)	22 (44.9)	
Islam	7 (1)	1 (14.3)	6 (85.7)		1 (14.3)	6 (85.7)		4 (66.7)	2 (33.3)	
Others	14 (2)	1 (7.1)	13 (92.9)		0 (0)	13 (100)		7 (53.8)	6 (46.2)	
**Living status**				0.054			0.330			0.679
Parents	337 (46.8)	49 (14.7)	284 (85.3)		67 (20.4)	262 (79.6)		223 (67.6)	107 (32.4)	
Father or mother	111 (15.4)	12 (11)	97 (89)		17 (15.3)	94 (84.7)		67 (61.5)	52 (38.5)	
Other relatives	65 (9)	12 (18.5)	53 (81.5)		14 (23)	47 (77)		39 (61.9)	24 (38.1)	
Alone	151 (21)	12 (8)	138 (92)		21 (14.4)	125 (85.6)		95 (64.2)	53 (35.8)	
Not with relatives	56 (7.8)	3 (5.4)	53 (94.6)		8 (14.3)	48 (85.7)		39 (69.6)	17 (30.4)	
**Family time activity (hours/week)**				0.126			0.168			0.389
< 2	240 (33.3)	20 (8.3)	220 (91.7)		37 (15.7)	199 (84.3)		149 (63.1)	87 (36.9)	
2–5	259 (35.9)	35 (13.8)	219 (68.2)		46 (18.2)	206 (81.8)		165 (64.4)	91 (35.6)	
6–10	121 (16.8)	19 (16)	100 (84)		29 (24.8)	88 (75.2)		79 (67.5)	38 (32.5)	
> 10	101 (14)	14 (13.9)	87 (86.1)		15 (15.2)	84 (84.8)		71 (724)	27 (22.6)	
**Average monthly family income (baht)**				0.001			0.268			0.622
< 10,000	92 (12.8)	19 (20.9)	73 (79.1)		20 (22.7)	68 (77.3)		58 (64.4)	32 (35.6)	
10,001–20,000	255 (31.4)	34 (15.2)	189 (84.8)		46 (20.6)	177 (79.4)		148 (67)	73 (33)	
20,001–30,000	121 (16.9)	17 (14.2)	103 (85.8)		20 (17)	98 (83)		72(61)	46 (39)	
> 30,000	279 (38.9)	18 (6.5)	259 (93.5)		41 (15.1)	230 (84.9)		185 (67.5)	89 (32.5)	
**Social media platform**				0.001 ^c^			0.015			0.862
Facebook	187 (32.7)	39 (20.9)	148 (79.1)		42 (23.2)	139 (76.8)		114 (61.6)	71 (38.4)	
Line	29 (5.1)	3 (10.3)	26 (89.7)		5 (17.2)	24 (82.8)		18 (66.7)	9 (33.3)	
Instagram	192 (33.6)	24 (12.6)	167 (87.4)		43 (23.1)	143 (76.9)		128 (67.4)	62 (32.6)	
YouTube	85 (14.9)	12 (14.3)	73 (85.7)		19 (22.4)	66 (77.6)		56 (65.9)	29 (34.1)	
Twitter	74 (13)	1 (1.4)	73 (98.6)		4 (5.5)	69 (94.5)		49 (68.1)	23 (31.9)	
Others	4 (0.7)	0 (0)	4 (100)		2 (50)	2 (50)		3 (75)	1 (25)	
**Social media usage (hours/day)**				<0.001			0.005			0.116
< 2	20 (2.8)	8 (42.1)	11 (57.9)		9 (45)	11 (55)		14 (73.7)	5 (26.3)	
2–4	190 (26.4)	16 (8.5)	173 (91.5)		26 (14)	160 (86)		128 (68.4)	59 (31.6)	
5–6	263 (36.6)	32 (12.3)	229 (87.7)		52 (20)	208 (80)		177 (68.6)	81 (31.4)	
> 6	246 (34.2)	31 (12.7)	213 (87.3)		39 (16.5)	197 (83.5)		144 (59.8)	97 (40.2)	
**Devices used to access social media**										
**Private laptop/computer**	350 (48.8)			<0.001			0.001			0.307
No		63 (17.3)	302 (82.7)		81 (22.7)	276 (77.3)		224 (67.4)	118 (32.6)	
Yes		25 (7.2)	323 (92.8)		45 (13.1)	299 (86.9)		218 (63.7)	124 (36.3)	
**Public computer**	23 (3.2)			0.639			0.099^c^			0.622
No		86 (12.4)	606 (87.6)		126 (18.6)	553 (81.4)		449 (65.8)	233 (34.2)	
Yes		2 (9.1)	20 (90.9)		1 (4.4)	22 (95.6)		14 (60.9)	9 (39.1)	
**Smartphone**	690 (96)			0.004^c^			0.175			0.875
No		9 (32.1)	19 (67.9)		8 (27.6)	21 (72.4)		18 (64.3)	10 (35.7)	
Yes		79 (11.5)	607 (88.5)		119 (17.7)	554 (82.3)		445 (65.7)	232 (34.3)	
**Tablet**	249 (34.6)			<0.001			0.020			0.926
No		81 (17.4)	384 (82.6)		94 (20.6)	363 (79.4)		302 (65.8)	157 (34.2)	
Yes		7 (2.8)	242 (97.2)		33 (13.5)	212 (86.5)		161 (65.4)	85 (34.6)	

^a^ percentage of the total ware represented by column and percentage of the Awareness, Perception, and Perpetration of Cyberbullying were presented by row.

^b^ Comparisons were performed by using Chi-squared tests for categorical variables and Mann-Whitney U tests for continuous variables.

^c^ Corrected *p*-values derived by using Fisher’s exact tests due to an expected frequencies violation in the Chi-squared test.

IQR, interquartile range.

### The undergraduates’ awareness of cyberbullying

Women were more aware of cyberbullying on social media than men (92.1% vs. 82.7%; *p* <0.001). The proportion of cyberbullying awareness was higher in students from other provinces than those from Chiang Mai (91.7% vs. 83.3%; *p* <0.001). The undergraduates’ awareness of cyberbullying depended on how much time they spent on social media, with the highest proportion comprising those who spent 2–4 hours per day and the lowest proportion comprising those who spent less than 2 hours per day (91.5% vs. 57.8%; *p* <0.001) (Table 2).

### The undergraduates’ perception of cyberbullying

Students who were from provinces other than Chiang Mai had a higher perception of cyberbullying than those who were from Chiang Mai (85.9% vs. 77.6%; *p* = 0.005). The highest usage of online media in which cyberbullying was perceived was Twitter (94.5%), followed by Line, YouTube, Instagram, Facebook, and others (82.8%, 77.6%, 76.9%, 76.8%, and 50%, respectively) (*p* = 0.015). Those who spent 2 hours or more a day on social media were more likely to perceive cyberbullying than those who spent less than 2 hours per day (>80% vs. 55%; *p* = 0.005) (Table 2).

### The undergraduates’ perpetration of cyberbullying

Men were more likely to perpetrate cyberbullying than women (39.9% vs. 29.4%; *p* = 0.003). Students in higher undergraduate years experienced more cyberbullying behavior, with the highest number being fifth-year students and higher (83.3%), followed by fourth-year (42.6%), third-year (34.5%), first-year (33.8%), and second-year (29.7%) (*p* = 0.020). There was also a difference between the bodyweights of those who had and had not experienced cyberbullying behavior (58 (IQR: 50.3–65) kg vs. 55 (IQR: 49–65) kg; *p* = 0.007). There was no difference in social media usage patterns between people who had and had not been subjected to cyberbullying behavior (Table 2).

### Comparison of the awareness, perception, and perpetration of cyberbullying between the high school and the undergraduate students

The proportions of awareness, perception, and perpetration of cyberbullying by the high school students and undergraduates were 82.4% vs. 87.7%, 79.8% vs. 82.0%, and 21.3% vs. 34.4%, respectively. Hence, it can be seen that awareness and perpetration of cyberbullying by the undergraduates were markedly higher than by the high school students. Most of the high school students and undergraduates (93.4% vs. 92.3%) received the information about cyberbullying via social media. The proportion of undergraduates who perceived cyberbullying due to their own experiences was higher than that of the high school students (48.2% vs. 30.8%, *p* <0.001). Moreover, the proportion of undergraduates who gained information on cyberbullying from teacher/school/training was also higher than that of the high school students (39.4% vs. 32.4%, *p* = 0.001). The proportion of high school students who informed their teachers/parents when facing cyberbullying was higher than that of the undergraduates (15.5% vs. 11.5%, *p* = 0.017), while the proportion of high school students who joined in the cyberbullying activities was lower (2.6 vs. 6.7, *p* <0.001). The main reason for cyberbullying by the high school and undergraduate students was the perpetrators’ aim to tease their targets, which was significantly higher in the undergraduates (67.6% vs. 82.5%, *p* <0.001). The proportions of undergraduates who have previously been victims, become a perpetrator (28.2% vs. 18.4%, *p* = 0.001), and forced to be a perpetrator (5.7% vs. 1.4%, *p* <0.001) were higher than those of the high school students (Table 3).

**Table 3:** Comparison of the awareness, perception, and perpetration of cyberbullying between the high school and undergraduate students.

Item (Number (%^a^))	Student Group		
	High School	Undergraduate	*P* ^b^
**Awareness**			
Know	2,181 (82.4)	626 (87.7)	0.001
Do not know	465 (17.6)	88 (12.3)	
**Channel of information** ^**b**^			
Social media	2,060 (93.4)	577 (92.3)	0.354
Own experience	679 (30.8)	301 (48.2)	<0.001
Teacher/School/Training	714 (32.4)	246 (39.4)	0.001
Others	42 (1.9)	10 (1.6)	0.620
**Perception**			
Never experienced	553 (20.2)	127 (18.0)	0.191
Experienced	2,099 (79.8)	577 (82.0)	
**Actions in response to cyberbullying** ^**c**^			
Ignore/Avoid	1,242 (58.7)	352 (60.5)	0.445
Dissuade the perpetrators	877 (41.5)	244 (41.8%)	0.880
Join the perpetration	55 (2.6)	39 (6.7)	<0.001
Inform teachers/parents	327 (15.5)	67 (11.5)	0.017
Collect evidence for the victims	401 (19.0)	119 (20.4)	0.437
Others	90 (4.3)	21 (3.6)	0.471
**Perpetration**			
Never done	2,042 (78.7)	464 (65.6)	<0.001
Ever done	552 (21.3)	243 (34.4)	
**Reason for cyberbullying perpetration** ^**c**^			
Aim to tease other people	446 (67.6)	203 (82.5)	<0.001
Have previously been a victim	122 (18.4)	69 (28.2)	0.001
Revenge on previous perpetrators	135 (20.2)	51 (20.8)	0.848
Coerced by other people	9 (1.4)	14 (5.7)	<0.001
Dislike of the victim	119 (18)	52 (21.2%)	0.271
Defamation by the victim	125 (18.8)	42 (17.6)	0.667
Other	22 (3.3)	10 (4.1)	0.580

^a^ percentage by column.

^b^ Comparisons were performed by using Chi-squared tests.

^c^ Multiple choice.

### Cyberbullying victimization by the participants

**Fig 1:**
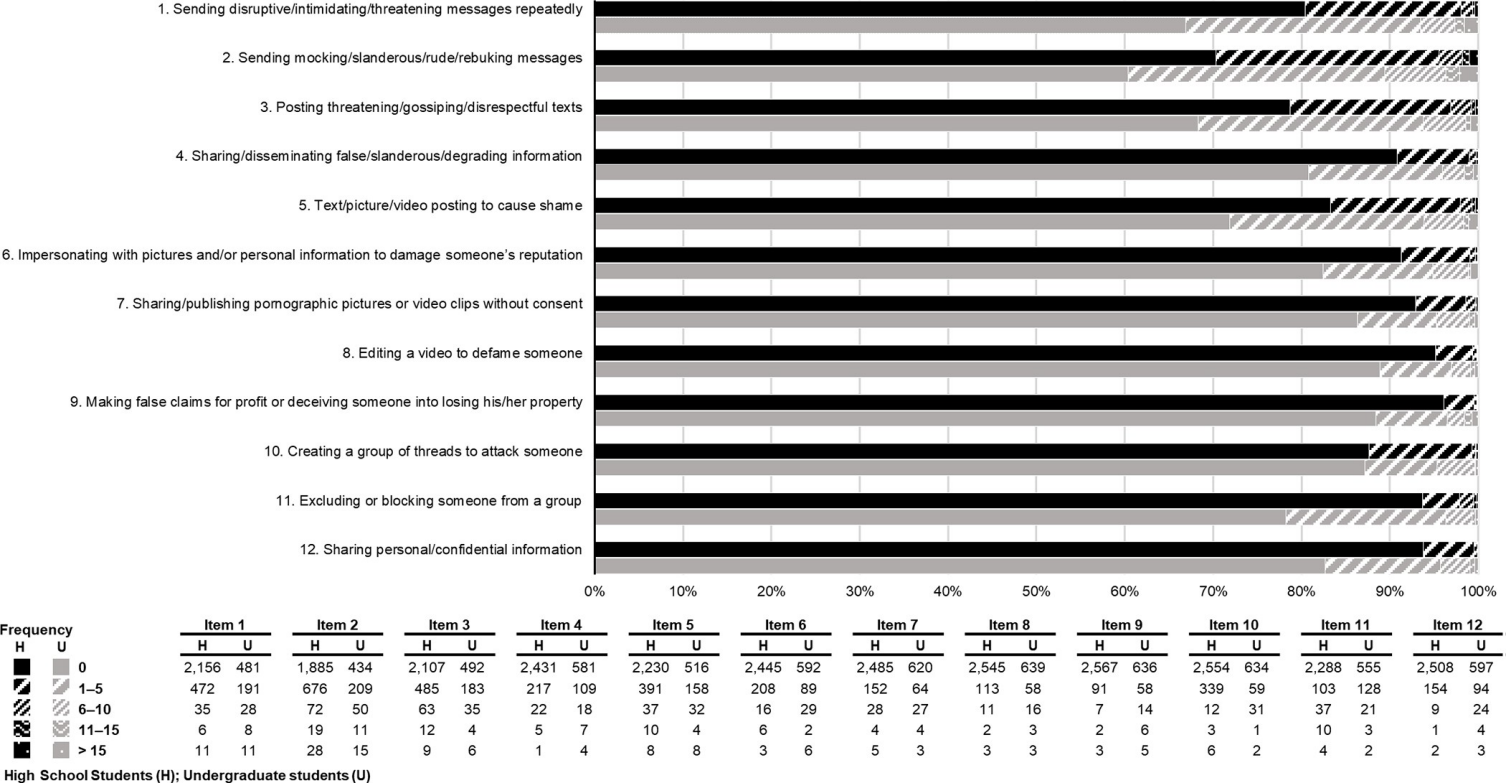
Frequency of cyberbullying victimization (N = 3,404).

Cyberbullying victimization activities perpetrated by the participants are summarized in Fig 1. The most commonly undertaken by the high school students was sending mocking/slanderous/rude/rebuking messages (29.6%), followed by posting threatening/ gossiping/disrespectful messages on social media (21.3%), sending disruptive/ intimidating/ threatening messages repeatedly (19.5%), and posting texts/pictures/videos to cause shame (16.5%). Most students who had been cyberbullied had been so 1 to 5 times (3.4–25.2%) whereas 50.8% of the respondents had never been cyberbullied.

Among the undergraduates, the most frequent cyberbullying victimization activity was sending mocking/slanderous/rude/rebuking messages (39.6%), followed by sending disruptive/intimidating/threatening messages repeatedly (33.1%), posting threatening/ gossiping/disrespectful messages (31.7%), posting texts/pictures/videos to cause shame (28.1%), and excluding or blocking an individual from a group (22.8%). Moreover, 63.5% of people had never been cyberbullied on social media of any kind.

### Dealing with cyberbullying by the participants

The majority (75.3%) of the high school students dealt with cyberbullying by avoiding leaving traces on social media, while the second most common way was spending time with a sympathetic friend (70.4%), never forwarding messages or images that hurt others when he/she was being cyberbullied (59.7%), and avoiding posting private information (56.8%). For occasional cyberbullying, most students discouraged the perpetrator (58.7%), followed by ignoring the cyberbully (57.4%) and trying not to think about being teased/cyberbullied (53.8%). In contrast, the least common ways to deal with cyberbullying were to seek help from the police or a psychiatrist (66.2%), inform the Internet service provider to stop publishing defamatory information (49.5%), and collect evidence of cyberbullying for retaliation (41.6%).

**Fig 2:**
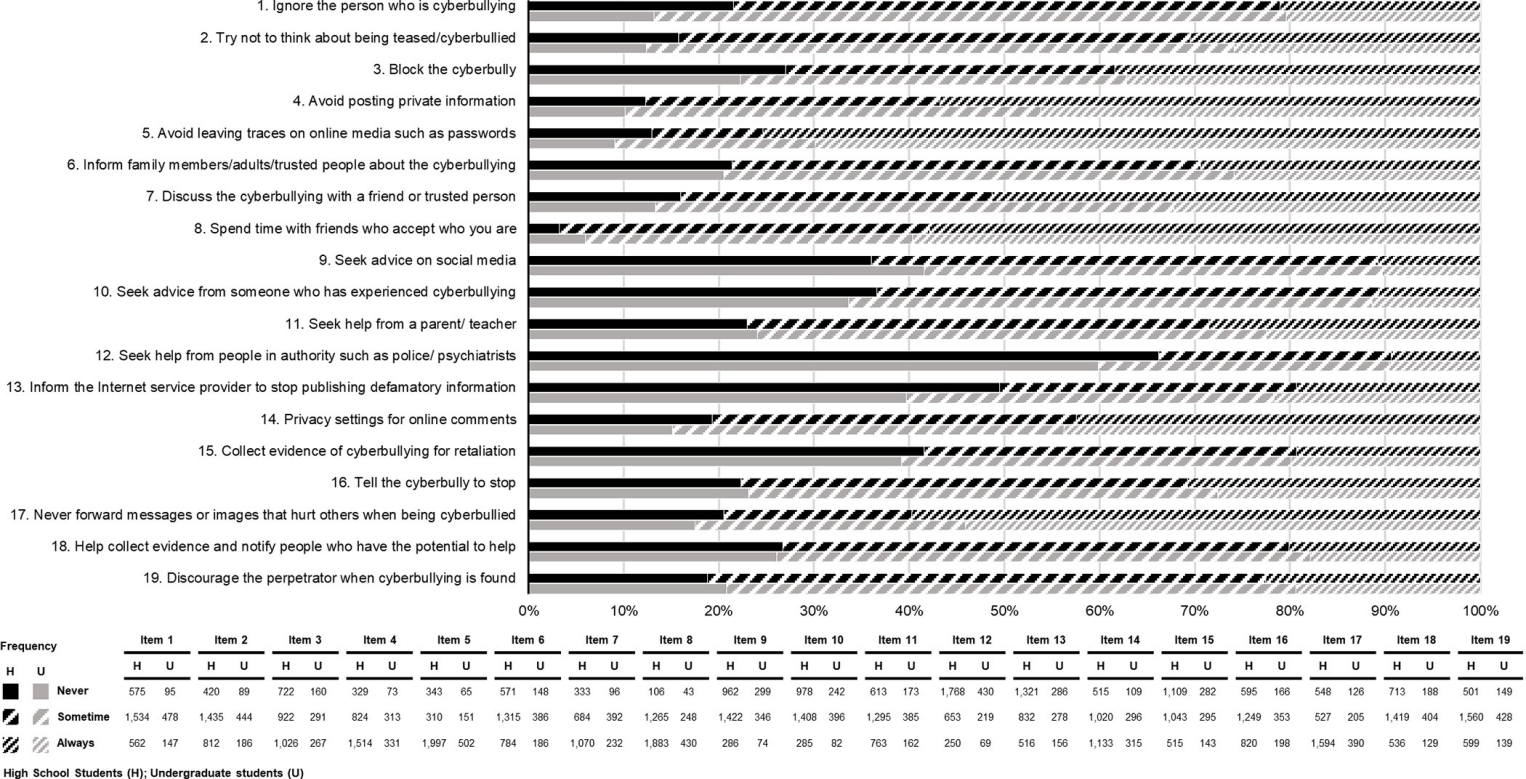
Dealing with cyberbullying (N = 3,404).

For the undergraduates, the most common countermeasures included avoiding leaving traces on online media such as passwords (69.9%), spending time with friends who accept you for who you are (59.6%), and not forwarding messages or images that hurt others while being cyberbullied (54.1%). The top three actions taken were ignoring the person carrying out the cyberbullying (66.4%), trying not to think about being teased/cyberbullied (61.7%), and discouraging the cyberbullying perpetrator (59.8%) (Fig 2).

## Discussion

Our findings reveal that half of the students (50.8% in high schools and 63.5% in universities) had been cyberbullied, which is higher than that reported in another study in Thailand in 2016 (one-third) [6]. The difference in the prevalence of cyberbullying among adolescents in Thailand between the present study and previous ones could be the result of differences in methodology, including operationalization of cyberbullying and the study population. In addition, this study was conducted during the COVID-19 pandemic with some restrictions such as online learning and staying at home. Thus, the probable increase in time spent on social media/the Internet and the stress of the lockdown measures could have increased the incidences of cyberbullying. Previously, researchers have stated that lockdown measures and school closures during the COVID-19 pandemic have substantially increased the online activity of children and adolescents globally, which has potentially increased the rate of cyberbullying [56]. According to a previous study on social media usage by children during the pandemic [57], the authors found that online content depicting violence has increased and a significant increase in abusive content during the stay-at-home restrictions.

The main factors that affect the three areas of cyberbullying studied (awareness, perception, and perpetration) are region, the people who you live with, the social media platform, time spent on social media, and tablet use. Of the three areas, we found that awareness of cyberbullying had the highest number of correlating factors. Thus, regardless of gender or personal behavior, awareness is key to becoming knowledgeable about cyberbullying.

The proportion of undergraduates with awareness of cyberbullying was significantly higher than for the high school students. The most common source of information about cyberbullying was social media for both high school and undergraduate students with no difference between these groups. However, the awareness of cyberbullying from their own experience or teachers/school/training was higher in the undergraduates than the high school students. Even though we did not record the length of time of experiencing cyberbullying, integration and training about cyberbullying and its effect on both the victims and perpetrators in a high school course might increase cyberbullying awareness and decrease its prevalence in the future. The findings from a previous study about classroom-based empathy training on cyberbullying in German schools [58] suggest that long-term intervention is more effective in reducing cyberbullying and promoting affective empathy. More than half of the high school students and undergraduates ignored or avoided cyberbullying after perceiving it. Interestingly, more undergraduates joined in perpetrating cyberbullying after perceiving it than high school students, which might be related to environmental changes, more freedom of action, and/or a lower propensity to inform teachers/parents in the undergraduate group. A previous study in Ghana [59] found that the percentage of undergraduates who perceived cyberbullying was not different from senior high school students (93.9% vs. 92.0%), which is consistent with our results. However, it was higher than the junior high school students (93.9% vs. 69.3%). In our study, most of the undergraduate and high school students stated that they perpetrated cyberbullying with the aim of teasing the victim, with the proportion of undergraduates doing so being higher than the high school students. Another interesting reason for cyberbullying perpetration was being coerced by other people, which was again higher among the undergraduates. These findings emphasize that informing about the effects of cyberbullying and cultivating empathy at high school could have an important role in reducing the instance of cyberbullying.

We found that more years of education in high school is linked to a higher incidence of cyberbullying behavior, which is inconsistent with Slonje and Smith’s [60] study who found that cyberbullying occurs most often among middle schoolers (grades 5 to 9). Moreover, Sirirassamee and Sirirassamee found that undergraduates in Thailand had a higher prevalence of cyberbullying than other groups, which is also inconsistent with our findings. This could have been because we only recruited students from Chiang Mai universities [16]. Nevertheless, it would be interesting to study whether different social conditions, regions, and/or educational levels affect the perception and awareness of cyberbullying differently. In the future, the study framework could be expanded to include these three variables to more widely cover perceptions and attitudes toward the problems of cyberbullying and cyber grooming [61].

In our study, gender had a significant effect in that females were cyberbullied more often than males and males were more often likely to cyberbully than females, possibly due to the pattern of online media use or socialization. This is consistent with the evidence for a relationship between cyberbullying and traditional bullying, as well as one between gender and the impact of Internet use on cyberbullying [8, 15, 62, 63]. In the Thai context, this could result from differences in behavior or ideas between genders or other related factors, such as upbringing, self-esteem, frustration, etc. In addition, the longer the time spent on social media, the higher the likelihood of cyberbullying or being cyberbullied. This is consistent with the findings from previous studies to identify factors associated with cyberbullying among Belgian [9] and Turkish adolescents [64]. The researchers discovered that prolonged Internet use, information technology proficiency, and owning a computer with Internet access were critical factors contributing to cyberbullying.

The findings from the present study reveal that the cyberbullying activities perpetrated by high school students and undergraduates were similar. The most popular was text messaging to either mock or harass (which directly affects the victim), which was used more often by undergraduates than high school students (39.6% vs. 29.6%, respectively), posting embarrassing images or texts (31.7% vs. 21.3%%, respectively), and sending disruptive/intimidating/threatening messages repeatedly (33.1% vs. 19.5%%, respectively) (which indirectly affects the victim); if they cannot be seen on the Internet or online, they will be scorned offline by others instead. Our findings show that most of the cyberbullying perpetrators did not consider that their actions would have serious consequences and only carried out cyberbullying because of wanting to tease their victims. This is useful information for the cyberbullying solution center, teachers, and parents to recognize how to make the students realize the effects of cyberbullying on the victims.

The actions taken by high school students and undergraduates to deal with cyberbullying were similar, including avoiding leaving traces of Internet usage and being with a trusted friend. However, the high school students only occasionally tried to dissuade the cyberbully, while the undergraduates more often ignored the cyberbully and tried not to think about being cyberbullied. These different methods might be related to the undergraduates being more experienced and psychologically resilient, and thus being more capable of emotional self-control and having a more grown-up mindset when confronting the problem [65]. Santos et al. [33] also mentioned that psychological resilience is a good protective factor against cyberbullying and other forms of victimization. In addition, we found that both the high school students and the undergraduates were reluctant to deal with cyberbullying by approaching the police or psychiatrists, which might be related to their reluctance to involve the authorities.

Although there is no specific law against cyberbullying in Thailand, the country’s legal framework does address cyberbullying activities under both criminal and cybercrime aspects to some extent [66]. Criminal law covers defamation, which is difficult to prove because it requires a third party to see or determine that what is said is not valid on the grounds of reality. Although distributing pornography is illegal and falls under the computer crime remit, it must be proven that an image accurately portrays the victim. Suwannakit [67] also referred to civil and commercial law to tackle the cyberbullying problem. Although it is illegal to deceive others and take their property, it is challenging to trace these incidences on the Internet. The most prominent types of cyberbullying identified in our study that might fall under the law as it stands include posting false information to defame people, disseminating pornographic videos, and impersonating others for profit. However, making and enforcing specific cyberbullying laws is required urgently to reduce the prevalence of cyberbullying in the future.

Although the law in Thailand is currently unenforceable for cyberbullying, we suggest practical guidelines for dealing with this phenomenon. For the US guidelines [68], educational institutions are encouraged to establish regulations on cyberbullying and define more explicit terms for it. Moreover, there is a system for centralized authorities to monitor and take responsibility for standardizing cyberbullying and taking measures to prevent it. In the Thai context, educational institutions should establish procedures and investigative methods, as well as provide evidence of cyberbullying and training on cyberbullying prevention for students. For decisive action, educational institutions should implement disciplinary measures for cyberbullying perpetrators, such as suspension from studies or expulsion, and will need to implement classroom-based interventions about empathy, safe use of the Internet, and avoiding cyberbullying. Moreover, initiatives to educate students on cyberbullying and workshops for both students and parents are required. Furthermore, undergraduates should be offered courses in avoiding being a victim of cyberbullying. We also found that the victims of cyberbullying rarely took legal action such as reporting the offense to their educational institute or asking for help from the police. Therefore, the solution center and other people involved in providing support to the cyberbullying victims should clarify the process of asking for help and offer possible solutions for mitigating the effects of the cyberbullying.

The strength of this study was the large number of high school student participants (both cyberbullying victims and perpetrators) to help reveal the effects of and reasons for cyberbullying. Another strength was that we compared cyberbullying characteristics between high school students and university undergraduates to investigate the effects of age, educational level, and lifestyle on cyberbullying. This study had some limitations. First, most of the participants from the high schools were from the northern and central regions of Thailand, and thus the study results might not be representative of the whole adolescent population in Thailand. However, we collected data from all regions of Thailand to adjust for the effect of cultural differences between the regions. Second, according to our aim, we focused on the comparison of cyberbullying between the undergraduates (most of whom were from the 1st and 2nd year at the Chiang Mai universities) and the high school students. However, we only enrolled undergraduates from the Chiang Mai province, so this sample might not be representative of the entire undergraduate population in Thailand. Thus, a nationwide undergraduate sample should be investigated to confirm whether the findings can be generalized for this population. Third, due to the nature of cross-sectional studies, although we could not conclude the cause-effect relationship of cyberbullying variables presenting significant differences between the groups, these findings could provide useful information for further investigation and reducing the incidence of cyberbullying in schools and universities. Fourth, the prevalence of cyberbullying perpetration might be lower than in reality due to reporting bias resulting from employing a self-reporting questionnaire. For instance, some respondents might not have recognized that their actions are related to cyberbullying. Including more examples of cyberbullying actions or more insightful questions that could be used to indirectly evaluate cyberbullying perpetration might reduce this issue. Next, the definition of cyberbullying is not explained directly by the questions concerning the awareness, perception, and perpetration of cyberbullying. Hence, understanding these terms could have varied among the participants and thus might have affected the validity of the main outcomes. To reduce this issue, as part of the data collection procedure, we explained the definition of cyberbullying and all of the outcomes to the staff members at the school and university so that they could provide this information to the participants before distributing the questionnaire. In addition, we provided further information related to cyberbullying, such as the channel of awareness, the list of cyberbullying actions, reactions to cyberbullying, and dealing with cyberbullying, in the subsequent sections of the questionnaire. These actions should have reduced variation in the understanding of cyberbullying by the participants and thus mitigated its effect on the main outcomes. However, the questions related to the quality or state of being aware (knowledge and understanding that something is happening or exists) or expression as a way of understanding or thinking to standardize the perception of cyberbullying should be conducted in a future study. Finally, some potential associated factors of the awareness, perception, and perpetration of cyberbullying such as psychological and physical well-being were not included in this study. In future studies, we need to add these and others so as to more comprehensively cover these three aspects of cyberbullying.

## Conclusions

Male and older students were more likely to perpetrate cyberbullying. Text messaging to either mock or harass followed by posting embarrassing texts or images and sending disruptive/intimidating/threatening messages repeatedly made up the majority of cyberbullying incidences involving the students. In addition to educating students and caregivers to increase awareness and reduce the incidence of cyberbullying, counseling by experts might help cyberbullying victims develop psychological resilience. Cyberbullying solution centers to assist the victims are essential, and providing a legal framework for cyberbullying in Thailand should be conducted as soon as possible to reduce the prevalence of cyberbullying.

## Supporting information

S1 Table Cronbach’s alpha coefficients for the cyberbullying victimization scale (N = 3,404). (DOCX)

S2 Table Exploratory factor analysis for the cyberbullying victimization scale (N = 3,404). (DOCX)

S3 Table Factor loading and fitting of indices for the cyberbullying victimization scale (N = 3,404). (DOCX)

S4 Table Cronbach’s alpha coefficients for the reaction to cyberbullying scale (N = 3,404). (DOCX)

S5 Table Exploratory factor analysis for the reaction to cyberbullying scale (N = 3,404). (DOCX)

S6 Table Factor loading and fitting of indices for the reaction to cyberbullying scale (N = 3,404). (DOCX)

S1 Dataset (XLSX)
