# Correction: Awareness, perception and perpetration of cyberbullying by high school students and undergraduates in Thailand

**DOI:** 10.1371/journal.pone.0315210

**Published:** 2024-12-04

**Authors:** The PLOS ONE Staff

There are errors in the author affiliations. The correct affiliations are as follows:

Salinee Thumronglaohapun^1,2^, Benchalak Maneeton^3^, Narong Maneeton^3^, Sasikarn Limpiti^4^, Natthaporn Manojai^5^, Jeerayut Chaijaruwanich^6^, Unyamanee Kummaraka^1,2^, Ruethaichanok Kardkasem^1^, Tanarat Muangmool^7^, Suttipong Kawilapat^1,3^, Kanokkarn Juntaping^1^, Patrinee Traisathit^1,2,8^, Pimwarat Srikummoon^1,2^

**1** Department of Statistics, Faculty of Science, Chiang Mai University, Chiang Mai, Thailand, **2** Data Science Research Center, Department of Statistics, Faculty of Science, Chiang Mai University, Chiang Mai, Thailand, **3** Department of Psychiatry, Faculty of Medicine, Chiang Mai University, Chiang Mai, Thailand, **4** Faculty of Mass Communication, Chiang Mai University, Chiang Mai, Thailand, **5** Mplus Foundation, Chiang Mai, Thailand, **6** Data Science Research Center, Department of Computer Science, Faculty of Science, Chiang Mai University, Chiang Mai, Thailand, **7** Department of Obstetrics and Gynecology, Faculty of Medicine, Chiang Mai University, Thailand, **8** Research Center in Bioresources for Agriculture, Industry and Medicine, Chiang Mai University, Chiang Mai, Thailand.

The publisher apologizes for the error.
