# Supplementary material for: Awareness, perception and perpetration of cyberbullying by high school students and undergraduates in Thailand
Source: PLoS One. 2022 Apr 29;17(4):e0267702. doi: 10.1371/journal.pone.0267702 (PMC9053786; doi:10.1371/journal.pone.0267702)
Supplement: S1 Table — (DOCX) [file pone.0267702.s001.docx]

**S1 Table. Cronbach’s Alpha Coefficients for the Cyberbullying Victimization Scale (N = 3,404).**

| **Item** | **Item-Total Correlation** | **Item-Rest Correlation** | **Average Interitem Covariance** | **Cronbach’s Alpha** |
| --- | --- | --- | --- | --- |
| 1. Sending disruptive/intimidating/threatening messages repeatedly | 0.6418 | 0.5342 | 0.0794 | 0.8586 |
| 2. Sending mocking/slanderous/rude/rebuking messages | 0.7075 | 0.5870 | 0.0735 | 0.8586 |
| 3. Posting threatening/gossiping/disrespectful texts | 0.7093 | 0.6154 | 0.0766 | 0.8523 |
| 4. Sharing/disseminating false/slanderous/degrading information | 0.7181 | 0.6547 | 0.0811 | 0.8509 |
| 5. Text/picture/video posting to cause shame | 0.6230 | 0.5178 | 0.0809 | 0.8594 |
| 6. Impersonating with pictures and/or personal information to damage someone’s reputation | 0.6167 | 0.5353 | 0.0838 | 0.8577 |
| 7. Sharing/publishing pornographic pictures or video clips without consent | 0.5508 | 0.4641 | 0.0860 | 0.8616 |
| 8. Editing a video to defame someone | 0.6190 | 0.5563 | 0.0861 | 0.8580 |
| 9. Making false claims for profit or deceiving someone into losing his/her property | 0.6214 | 0.5577 | 0.0859 | 0.8577 |
| 10. Creating a group of threads to attack someone | 0.6678 | 0.6075 | 0.0844 | 0.8551 |
| 11. Excluding or blocking someone from a group | 0.6734 | 0.5899 | 0.0804 | 0.8539 |
| 12. Sharing personal/confidential information | 0.6692 | 0.6081 | 0.0842 | 0.8549 |
| **Test scale** | | | 0.0819 | 0.8670 |
