# Supplementary material for: Awareness, perception and perpetration of cyberbullying by high school students and undergraduates in Thailand
Source: PLoS One. 2022 Apr 29;17(4):e0267702. doi: 10.1371/journal.pone.0267702 (PMC9053786; doi:10.1371/journal.pone.0267702)
Supplement: S2 Table — (DOCX) [file pone.0267702.s002.docx]

**S2 Table. Exploratory Factor Analysis for the Cyberbullying Victimization Scale (N = 3,404).**

| **Factor** | **Eigenvalue** | **Difference** | **Proportion** | **Cumulative** |
| --- | --- | --- | --- | --- |
| **Factor1** | **4.5393** | **3.9881** | **0.9830** | **0.9830** |
| Factor2 | 0.5512 | 0.3601 | 0.1194 | 1.1023 |
| Factor3 | 0.1912 | 0.0740 | 0.0414 | 1.1437 |
| Factor4 | 0.1172 | 0.0859 | 0.0254 | 1.1691 |
| Factor5 | 0.0313 | 0.0481 | 0.0068 | 1.1759 |
| Factor6 | -0.0169 | 0.0387 | -0.0036 | 1.1722 |
| Factor7 | -0.0555 | 0.0543 | -0.0120 | 1.1602 |
| Factor8 | -0.1099 | 0.0058 | -0.0238 | 1.1364 |
| Factor9 | -0.1157 | 0.0302 | -0.0251 | 1.1113 |
| Factor10 | -0.1459 | 0.0176 | -0.0316 | 1.0798 |
| Factor11 | -0.1635 | 0.0413 | -0.0354 | 1.0444 |
| Factor12 | -0.2048 | . | -0.0444 | 1.0000 |
