# Supplementary material for: Awareness, perception and perpetration of cyberbullying by high school students and undergraduates in Thailand
Source: PLoS One. 2022 Apr 29;17(4):e0267702. doi: 10.1371/journal.pone.0267702 (PMC9053786; doi:10.1371/journal.pone.0267702)
Supplement: S3 Table — (DOCX) [file pone.0267702.s003.docx]

**S3 Table. Factor Loading and Fitting of Indices for the Cyberbullying Victimization Scale (N = 3,404).**

| **Item** | **Factor Loading** | **Uniqueness** |
| --- | --- | --- |
| 1 | 0.5471 | 0.7007 |
| 2 | 0.6032 | 0.6361 |
| 3 | 0.6441 | 0.5852 |
| 4 | 0.6887 | 0.5257 |
| 5 | 0.5512 | 0.6962 |
| 6 | 0.5995 | 0.6406 |
| 7 | 0.4996 | 0.7504 |
| 8 | 0.6115 | 0.6261 |
| 9 | 0.6343 | 0.5976 |
| 10 | 0.6746 | 0.5450 |
| 11 | 0.6400 | 0.5904 |
| 12 | 0.6583 | 0.5666 |
| **CFI** | **0.876** |  |
| **TLI** | **0.849** |  |
| **RMSEA** | **0.097** |  |

CFI, Comparative fit index, TLI, Tucker-Lewis index; RMSEA, Root mean squared error of approximation
