# Supplementary material for: Awareness, perception and perpetration of cyberbullying by high school students and undergraduates in Thailand
Source: PLoS One. 2022 Apr 29;17(4):e0267702. doi: 10.1371/journal.pone.0267702 (PMC9053786; doi:10.1371/journal.pone.0267702)
Supplement: S4 Table — (DOCX) [file pone.0267702.s004.docx]

**S4 Table. Cronbach’s Alpha Coefficients for the Reaction to Cyberbullying Scale (N = 3,404).**

| **Item** | **Item-Total Correlation** | **Item-Rest Correlation** | **Average Interitem Covariance** | **Cronbach’s Alpha** |
| --- | --- | --- | --- | --- |
| 1. Ignore the person who is cyberbullying | 0.3551 | 0.2741 | 0.1317 | 0.8638 |
| 2. Try not to think about being teased/cyberbullied | 0.4601 | 0.3843 | 0.1284 | 0.8600 |
| 3. Block the cyberbully | 0.5356 | 0.4497 | 0.1238 | 0.8578 |
| 4. Avoid posting private information | 0.5576 | 0.4856 | 0.1246 | 0.8562 |
| 5. Avoid leaving traces on online media such as passwords | 0.5671 | 0.4965 | 0.1244 | 0.8558 |
| 6. Inform family members/adults/trusted people about the cyberbullying | 0.6167 | 0.5505 | 0.1225 | 0.8536 |
| 7. Discuss the cyberbullying with a friend or trusted person | 0.5071 | 0.4102 | 0.1240 | 0.8602 |
| 8. Spend time with friends who accept who you are | 0.4298 | 0.3600 | 0.1301 | 0.8608 |
| 9. Seek advice on social media | 0.3633 | 0.2830 | 0.1314 | 0.8635 |
| 10. Seek advice from someone who has experienced cyberbullying | 0.5258 | 0.4575 | 0.1266 | 0.8574 |
| 11. Seek help from a parent/ teacher | 0.5955 | 0.5263 | 0.1231 | 0.8546 |
| 12. Seek help from people in authority such as police/ psychiatrists | 0.5263 | 0.4554 | 0.1262 | 0.8574 |
| 13. Inform the Internet service provider to stop publishing defamatory information | 0.6016 | 0.5247 | 0.1214 | 0.8545 |
| 14. Privacy settings for online comments | 0.5726 | 0.4970 | 0.1233 | 0.8557 |
| 15. Collect evidence of cyberbullying for retaliation | 0.5884 | 0.5142 | 0.1226 | 0.8550 |
| 16. Tell the cyberbully to stop | 0.6247 | 0.5578 | 0.1218 | 0.8532 |
| 17. Never forward messages or images that hurt others when being cyberbullied | 0.5838 | 0.5036 | 0.1220 | 0.8555 |
| 18. Help collect evidence and notify people who have the potential to help when others are being cyberbullied | 0.6168 | 0.5535 | 0.1230 | 0.8537 |
| 19. Discourage the perpetrator when cyberbullying is found | 0.5766 | 0.5126 | 0.1250 | 0.8554 |
| **Test scale** | | | 0.1251 | 0.8636 |
