# Supplementary material for: Awareness, perception and perpetration of cyberbullying by high school students and undergraduates in Thailand
Source: PLoS One. 2022 Apr 29;17(4):e0267702. doi: 10.1371/journal.pone.0267702 (PMC9053786; doi:10.1371/journal.pone.0267702)
Supplement: S5 Table — (DOCX) [file pone.0267702.s005.docx]

**S5 Table. Exploratory Factor Analysis for the Reaction to Cyberbullying Scale (N = 3,404).**

| **Factor** | **Eigenvalue** | **Difference** | **Proportion** | **Cumulative** |
| --- | --- | --- | --- | --- |
| **Factor1** | **5.0341** | **3.5786** | **0.7420** | **0.7420** |
| **Factor2** | **1.4554** | **0.8635** | **0.2145** | **0.9566** |
| Factor3 | 0.5920 | 0.1544 | 0.0873 | 1.0438 |
| Factor4 | 0.4376 | 0.1049 | 0.0645 | 1.1084 |
| Factor5 | 0.3327 | 0.1078 | 0.0490 | 1.1574 |
| Factor6 | 0.2249 | 0.1187 | 0.0331 | 1.1905 |
| Factor7 | 0.1062 | 0.0565 | 0.0156 | 1.2062 |
| Factor8 | 0.0496 | 0.0392 | 0.0073 | 1.2135 |
| Factor9 | 0.0104 | 0.0267 | 0.0015 | 1.2150 |
| Factor10 | -0.0163 | 0.0507 | -0.0024 | 1.2126 |
| Factor11 | -0.0670 | 0.0137 | -0.0099 | 1.2028 |
| Factor12 | -0.0807 | 0.0523 | -0.0119 | 1.1909 |
| Factor13 | -0.1330 | 0.0064 | -0.0196 | 1.1713 |
| Factor14 | -0.1394 | 0.0170 | -0.0206 | 1.1507 |
| Factor15 | -0.1565 | 0.0376 | -0.0231 | 1.1276 |
| Factor16 | -0.1941 | 0.0064 | -0.0286 | 1.0990 |
| Factor17 | -0.2005 | 0.0281 | -0.0295 | 1.0695 |
| Factor18 | -0.2286 | 0.0141 | -0.0337 | 1.0358 |
| Factor19 | -0.2427 | . | -0.0358 | 1.0000 |
