# Supplementary material for: Awareness, perception and perpetration of cyberbullying by high school students and undergraduates in Thailand
Source: PLoS One. 2022 Apr 29;17(4):e0267702. doi: 10.1371/journal.pone.0267702 (PMC9053786; doi:10.1371/journal.pone.0267702)
Supplement: S6 Table — (DOCX) [file pone.0267702.s006.docx]

**S6 Table. Factor Loading and Fitting of Indices for the Reaction to Cyberbullying Scale (N = 3,404).**

| **Item** | **Factor 1^a^** | **Factor 2^a^** | **Uniqueness** |
| --- | --- | --- | --- |
| 1 |  | 0.5521^c^ | 0.6929 |
| 2 |  | 0.5847^c^ | 0.6527 |
| 3 | 0.3110 | 0.3813^c^ | 0.7579 |
| 4 |  | 0.6632^c^ | 0.5335 |
| 5 |  | 0.6895^c^ | 0.5000 |
| 6 | 0.4622^b^ | 0.3824 | 0.6402 |
| 7 | 0.3068^b^ | 0.3334 | 0.7947 |
| 8 |  | 0.3301^c^ | 0.8367 |
| 9 | 0.4541^b^ |  | 0.7898 |
| 10 | 0.5750^b^ |  | 0.6657 |
| 11 | 0.5906^b^ |  | 0.6169 |
| 12 | 0.6382^b^ |  | 0.5927 |
| 13 | 0.5975^b^ |  | 0.6149 |
| 14 | 0.3749 | 0.3771^c^ | 0.7173 |
| 15 | 0.5902^b^ |  | 0.6265 |
| 16 | 0.5783^b^ |  | 0.6029 |
| 17 |  | 0.5083^c^ | 0.6518 |
| 18 | 0.6110^b^ |  | 0.5791 |
| 19 | 0.5537^b^ |  | 0.6443 |
| **CFI** | **0.792** | **0.866** |  |
| **TLI** | **0.740** | **0.812** |  |
| **RMSEA** | **0.123** | **0.108** |  |

CFI, Comparative fit index, TLI, Tucker-Lewis index; RMSEA, Root mean squared error of approximation

^a^ Items with factor loading <0.3 are presented as blanks

^b^ items 6, 7, 9, 10, 11, 12, 13, 15, 16, 18, and 19 are included subscale 1, seeking support from other people

^c^ items 1, 2, 3, 4, 5, 8, 14, and 17 are included in subscale 2, disregarding and preventing cyberbullying
